# Growth patterns of preterm and small for gestational age children during the first 10 years of life

**DOI:** 10.3389/fnut.2024.1348225

**Published:** 2024-02-26

**Authors:** Phuong Thi Nguyen, Phuong Hong Nguyen, Lan Mai Tran, Long Quynh Khuong, Son Van Nguyen, Melissa F. Young, Usha Ramakrishnan

**Affiliations:** ^1^Department of Pediatric, Thai Nguyen University of Medicine and Pharmacy, Thai Nguyen, Vietnam; ^2^Department of Nutrition, Diets, and Health, International Food Policy Research Institute, Washington, DC, United States; ^3^Hubert Department of Global Health, Emory University, Atlanta, GA, United States; ^4^Department of Biostatistics, Epidemiology, and Informatics, University of Pennsylvania, Philadelphia, PA, United States

**Keywords:** preterm, small for gestational age, growth pattern, velocity, overweight/obesity, stunting, Vietnam

## Abstract

**Background:**

Preterm and small for gestational age (SGA) remain significant public health concerns worldwide. Yet limited evidence exists on their growth patterns during childhood from low-or middle-income countries.

**Objectives:**

We investigated the postnatal growth patterns of preterm and SGA compared to term appropriate for gestational age (AGA) children from birth to 10–11y, and examined the impact of birth status on child nutritional status during the school age years.

**Methods:**

Children born to women who participated in a double-blinded randomized controlled trial of preconception micronutrient supplementation in Vietnam were classified into three groups: preterm AGA (*n* = 130), full-term SGA (*n* = 165) and full-term AGA (*n* = 1,072). Anthropometric data (weight and height) were collected prospectively at birth, 3, 6, 12, 18, 24 months and at 6–7 and 10–11y. We used ANOVA and multiple regression models to examine the differences in growth patterns from birth to 10–11y as well as child undernutrition and overnutrition by birth status.

**Results:**

Children who were born preterm exhibited rapid postnatal growth, but still had lower HAZ at 1y and 2y and showed catch up to the AGA group at 6y. Compared to those born AGA, SGA infants had higher risk of thinness (BMIZ < −2) at 2y and 6y (adjusted Odds Ratio, AOR [95% CI] 2.5 [1.0, 6.1] and 2.6 [1.4, 4.6], respectively); this risk reduced at 10–11y (1.6 [0.9, 2.8]). The risk of stunting (HAZ < −2) was also 2.4 [1.5, 3.8] and 2.3 times [1.2, 4.1] higher in SGA than AGA group at ages 2y and 6–7y, respectively, with no differences at 10y. Although preterm children had higher rates of thinness and stunting at 2y compared to AGA children, these differences were not statistically significant. No associations were found between preterm or SGA and overweight /obesity at age 10–11y.

**Conclusion:**

Children who were born term-SGA continued to demonstrate deficits in weight and height during childhood whereas those born preterm showed catch-up growth by age 6–7y. Additional efforts to reduce the burden of these conditions are needed, particularly during school-age and early adolescents when children are exposed to challenging environments and have higher demands for nutrition.

## Introduction

Preterm (birth before 37 weeks of gestation) and small for gestational age (SGA, birthweight less than 10th percentile for gestational age) are significant public health concerns worldwide. Preterm and SGA are not only the leading causes of mortality and morbidity among children aged under 5 years, but also have long-term consequences during childhood and later in life, including childhood undernutrition and illness, developmental loss, adult diseases and reduced human capital (1). It is estimated that 15 million infants were born preterm (11% of livebirths) each year, with 81% of them residing in Asia and sub-Saharan Africa (2). In addition, 32.4 million infants were born SGA (27% of livebirths) with two thirds of them being born in Asia (3, 4).

Previous research has documented that the growth patterns of preterm and SGA children are different from that of term appropriate for gestational age (AGA) children (5, 6). Although the mechanisms leading to preterm birth and SGA are different (7), they share many risk factors and consequences. Infants born preterm are typically immature and may be low birth weight because they are born early but may not have experienced fetal growth restriction which is typical of those born SGA (8, 9). Both preterm and SGA children often experience higher growth velocity compared to full term AGA children (8) especially in the first 6 months (10). Although previous literature has reported catchup growth among term SGA infants (11–13), the exact timing catch up growth and the potential continued effects of birth phenotype on growth faltering into early childhood remain unclear (6).

While there is ample evidence on growth patterns of preterm and SGA children in early childhood (10, 12, 14–17), limited evidence exists on growth during school age years and early adolescence. Growth patterns during this period are highly heterogenous and influenced by parental height (18, 19), illnesses, nutrition, and physical activities during childhood (3, 6). Findings from a study in Denmark showed that children born SGA demonstrated continuous catch-up growth until 6 years of age and the pattern of growth was related to child feeding (20). Other studies reporting on further growth into adolescence show incomplete catch-up growth and persistence of stunting especially in those born SGA. For example, a study from USA showed that healthy preterm infants caught up in height by 8 years of age, while those born SGA group remained shorter at age 12 years (21). Similarly, catch-up growth was seen by age 10 years among children born very preterm AGA in a prospective study of a nationally representative sample of Dutch children, but not among those born SGA (22).

Although catch-up growth is very important among preterm and SGA children, rapid catch-up growth might increase their risk for obesity (16, 23) and cardiometabolic diseases such as cardiovascular disease type 2 diabetes mellitus and other metabolic disorders during adulthood (24, 25). Establishing optimal growth patterns or healthy catch-up is needed to minimize the long-term risk. Most evidence on growth patterns and catch-up growth, however, comes from upper-middle- or high-income countries (11, 23, 26) while information on growth patterns of growth of preterm and SGA from low-or middle-income countries are scant with shorter follow-up time.

We have the unique opportunity to examine the growth patterns of preterm and SGA children using prospectively collected data from a well-designed longitudinal study that spans from preconception, during pregnancy, infancy through the school age years, from a low-middle income country setting, i.e., Vietnam. Vietnam is located in Southeast Asia where the rate of preterm birth ranges from 5 to 9% and the rate of SGA infants ranges from 10 to 20% (27). The objectives of this study were to (1) investigate postnatal growth patterns of preterm and SGA compared to term appropriate for gestational age (AGA) children from birth to 10–11y, and (2) to examine the impact of birth status on nutritional status (thinness, stunting, and overweight/obesity) during the school age years.

## Methods

### Data sources and study population

**Figure 1:**
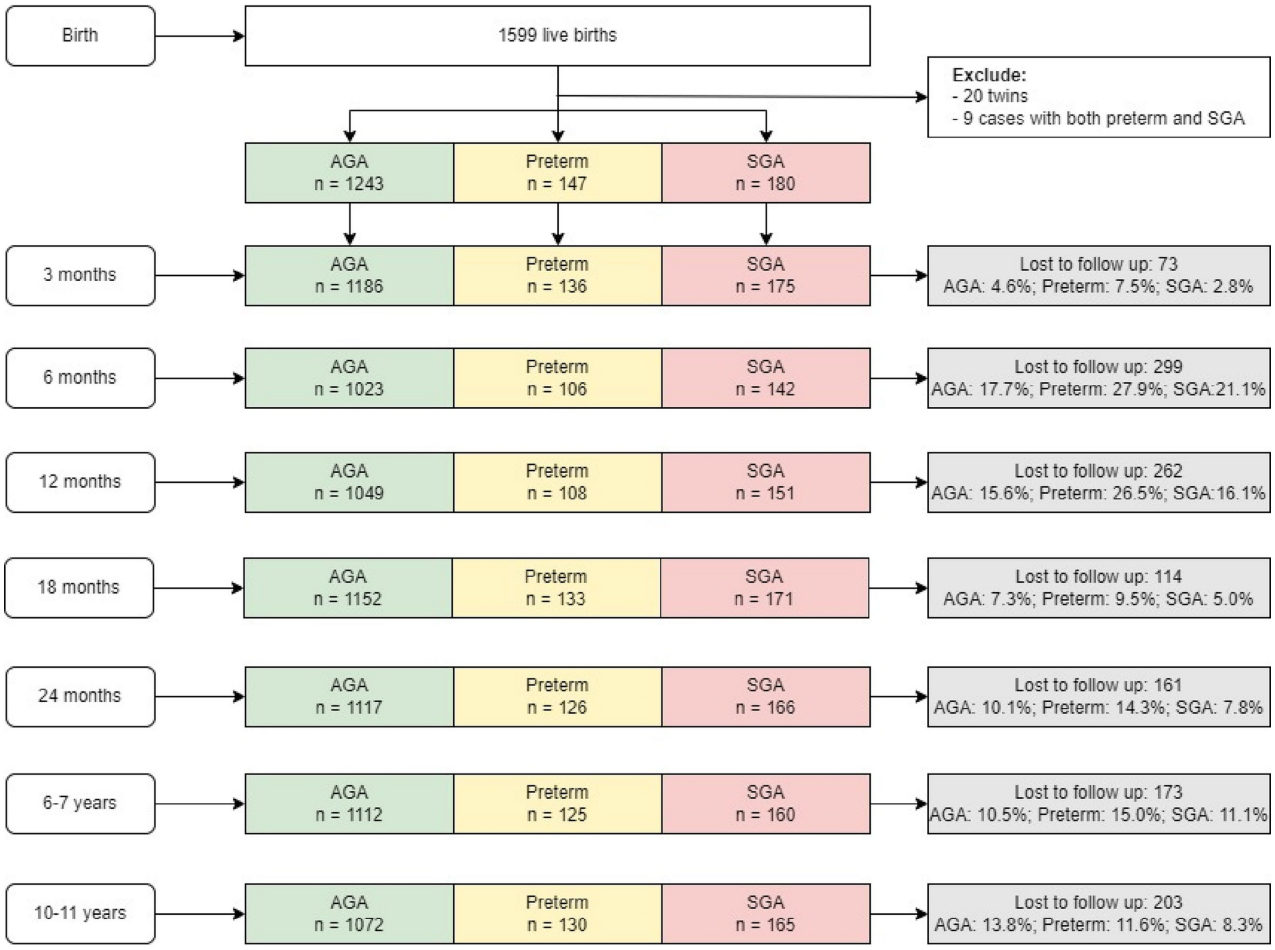
Sample flow. AGA, appropriate for gestational age; SGA, small for gestational age.

Children in this study are offspring of women who participated in a double-blind randomized controlled trial (PRECONCEPT; NCT: 01665378), which evaluated the effects of preconception micronutrient supplementation on maternal and child health outcomes (28). Details of the parent study have previously been published (28). Briefly, 5,011 women of reproductive age were randomly assigned to receive weekly supplements containing either 2,800 μg folic acid (FA), 60 mg iron and 2,800 μg FA (IFA), or multiple micronutrients (MM) containing the same amount of IFA, from baseline until conception, followed by daily prenatal supplements containing 60 mg iron and 400 μg FA until delivery. Women were followed prospectively to identify pregnancies and evaluate birth outcomes; 1,813 women conceived between 2012 and 2014 and 1,599 had live births. Live births were followed at 3 mo, 6 mo, and at 1, 2, 6–7, and 10–11y (Figure 1) with follow-up rates of 96, 82, 94, 89, and 87%, respectively. Findings on the main outcomes of the trials have been published elsewhere (29, 30). The current analysis includes all children with available data on birth weight, gestational age and anthropometry data at different periods. Participants were divided into the following three groups: (1) Pre-term newborn: gestational age < 37 weeks; (2) Full-term SGA newborn: gestational age ≥ 37 weeks and birth weight below the 10th percentile; (3) Full-term AGA newborn (gestational age ≥ 37 weeks and birthweight above the 10th percentile). There were only nine preterm SGA children—therefore, we excluded them from the analysis.

### Preterm and SGA

Gestational age was estimated based on the last menstrual period (LMP) which was obtained prospectively by village health workers during biweekly home visits. Gestational age at delivery was calculated by subtracting a woman’s LMP from her delivery date. In case the last menstrual period-based gestational age was unknown or inconsistent, we adopted the ultrasound estimates which was considered the gold standard in clinical maturity estimate (31). Preterm was defined as a birth that occurs before 37 completed weeks (less than 259 days) of pregnancy.

Birth weight was measured as early as possible within 7 days after birth using standard procedures (32, 33). Naked weight was measured using a UNICEF Beam type scale for infants and readings were made to the nearest 10 g (34). All measurements are obtained in duplicate by trained district hospital staff. SGA was defined as a birth weight below the 10th percentile for gestational age and sex based on the multi-country INTERGROWTH-21st Project (35).

### Anthropometric measurements and child growth indicators

Child weight and length/height were measured at birth, 3 months, 6 months, 12 months, 18 months, 2y, 6–7y and 10–11y by trained and standardized field staff using standard methods (33). Child weight was measured using electronic weighing scales precise to 10 g. Child length in the supine position (from birth to 2 years) and child standing height (after 2 years) were measured using collapsible length boards, which were precise to 1 mm. The average of duplicate measurements of height and weight were then converted into height-for-age z-scores (HAZ), and body-mass-index for-age z-scores (BMIZ) according to INTERGROWTH-21st for postnatal growth of preterm infants (36), 2006 WHO child growth standards (37) and growth reference data for 5–19 years (38). Stunting was defined as HAZ below −2 Z-score. Thinness was defined as BMIZ below −2 Z-score and overweight/obese as BMIZ above 1 Z-score (38). Growth velocity (grams/month or centimeters/month) (39) was assessed by evaluating growth in the intervals between birth and 3 mo, 6 mo, 12 mo, 18 mo, 2y, 6–7y and 10–11y.

### Covariables

Other variables were collected at child, maternal and household levels. At the child level, we collected information on sex and child feeding during the first 2 years (early initiation of breastfeeding, exclusive breastfeeding at 3 months and minimum dietary diversity at 1 year). At the maternal levels, we collected information on maternal age, education, occupation, parity, and maternal nutritional status. Maternal pre-pregnancy weight and height were measured at enrollment (preconception) in Community Health Centers by trained staff using standard procedures (32, 33). Pre-pregnancy body mass index (BMI) was calculated as weight/height^2^ (kg/ m^2^). Maternal underweight was defined as BMI <18.5 kg/ m^2^ and overweight as BMI >23 kg/ m^2^ based on suggested cut-off for Asian population (40). At the household level, we collected information on household socio-economic status (SES) which was calculated using a principal components analysis of assets and services, including house and land ownership, housing quality, access to services (electricity, gas, water, and sanitation services), and household assets (productive assets, durable goods, animals, and livestock). The first component derived from component scores were used to divide household SES into quartiles (41, 42).

### Statistical analysis

Normality of the continuous outcome variables was assessed using the Kolmogorov–Smirnov test. Descriptive analyses (means, standard deviations, percent) were used to report characteristics of the study population by three birth condition groups (preterm, SGA and AGA), using chi-square and one-way ANOVA where appropriate. We also compared baseline characteristics of study participants in the final analytic sample and those lost to follow-up.

To examine the differences in growth pattern (weight, height, BMIZ, HAZ) and growth velocity, we first used the ANOVA to test for overall differences among three birth condition groups, followed by Bonferroni post-hoc correction for pair-wise comparison. Multiple logistic regression models were used to estimate relationships between SGA vs. AGA and preterm vs. AGA status at birth on child undernutrition (stunting and thinness) and overnutrition (overweight/ obesity) at 2y, 6–7y and 10–11y. All models adjusted for potential confounding factors including at child (sex, current age at follow-up), maternal and household levels. All data analyses were performed using STATA version 17. Results were considered significant when *p* < 0.05.

### Ethical approval

The study was approved by the Ethical Committee of Institute of Social and Medicine Studies in Vietnam and Emory University’s Institutional Review Board, Atlanta, Georgia, United States. The trial was registered in the US Clinical Trials registry (identification number NCT01665378). Written informed consent was obtained from all study participants.

## Results

### Participants flow

We successfully followed 87% of the birth cohort up to 10–11 years of age (*n* = 1,391 out of 1,599 live births) (Figure 1). We lost 13% of the cohort because of migration out of the study area (*n* = 123), dropouts (*n* = 70) or child mortality (*n* = 10). The final sample at 10–11 y comprised 130 preterm, 165 SGA and 1,072 AGA children. The final analytic sample was similar on most child characteristics to those with missing data (Supplementary Table S1). However, women in the missing sample tended to be younger, have higher level of education, and live in household with higher SES, compared to those in the analytic sample.

### Maternal and child characteristics, by birth condition

The characteristics of the study subjects are presented in Table 1. At birth, women were on average 26 years and > 90% gave birth to the second child. There were no significant differences in maternal age, education or occupation but mothers of SGA infants were significantly shorter (151.7 cm) compared to those in the preterm (152.3 cm) and AGA group (153.0 cm) (*p* < 0.05). The prevalence of preconception underweight among mothers was also highest in the SGA group compared to the other two groups (39.7% in SGA vs. 32.5% in preterm and 29.7% in AGA group, *p* < 0.05).

**Table 1:** Characteristics of study sample born preterm, SGA, and AGA.

	AGA (*n* = 1243)	Preterm (*n* = 147)	SGA (*n* = 180)
Mother characteristics			
Mother’s age at birth	26.0 (4.3)	25.8 (4.8)	25.3 (4.1)
Mother education			
Completed primary school	91 (7.3)	19 (12.9)	16 (8.9)
Completed secondary school	666 (53.6)	85 (57.8)	98 (54.4)
Completed high school	324 (26.1)	30 (20.4)	47 (26.1)
College or higher	162 (13.0)	13 (8.8)	19 (10.6)
Occupation			
Work as farmers	976 (78.5)	120 (81.6)	154 (85.6)
Other jobs	267 (21.5)	27 (18.4)	26 (14.4)
Parity			
1 child	51 (4.8)	10 (8.1)	10 (7.1)
≥2 children	1012 (95.2)	113 (91.9)	130 (92.9)
Mother’s height	153.0 (5.0)	152.3 (5.0)	151.7 (5.4)**
BMI			
Mother underweight (BMI<18.5)	368 (29.7)	46 (31.5)	71 (39.7)*
Mother overweight (BMI ≥23)	84 (6.8)	7 (4.8)	7 (3.9)
Household characteristics			
Socio-economic status			
High	408 (32.9)	50 (34.0)	74 (41.1)
Middle	410 (33.0)	46 (31.3)	61 (33.9)
Low	423 (34.1)	51 (34.7)	45 (25.0)
Child characteristics			
Birthweight (g)	3183 (389)	2874 (460)	2571 (264)***
Birth length, cm	49.3 (2.8)	48.7 (2.9)	47.0 (3.1)***
Gestational age (weeks)	39.5 (1.3)	34.9 (1.7)	40.5 (1.7)***
Sex (female)	622 (50.0)	65 (44.2)	87 (48.3)
Child feeding			
Early initiation of breastfeeding	615 (52.4)	52 (38.0)	90 (57.0)**
Exclusive breastfeeding at 5 months	633 (58.9)	72 (56.3)	96 (62.7)
Dietary diversity at 1y	705 (69.5)	70 (64.8)	104 (75.4)
Minimum dietary diversity at 2y	127 (59.6%)	11 (55.0%)	20 (64.5%)
Minimum dietary diversity at 6–7y	859 (69.1)	94 (63.9)	124 (68.9)
Child morbidity at 1y			
ARI	596 (58.7)	68 (63.0)	82 (59.4)
Diarrhea	130 (12.8)	20 (18.5)	22 (16.1)
Child morbidity at 2y			
ARI	539 (48.1)	64 (50.8)	91 (57.2)
Diarrhea	62 (5.5)	10 (7.9)	11 (7.0)
Child morbidity at 6–7y			
ARI	319 (29.2)	35 (28.5)	62 (40.8)*
Diarrhea	8 (0.7)	3 (2.4)	5 (3.3)**

Values are percentage or mean (SD). Significant difference among three groups by ANOVA test: ^*^p < 0.05, ^**^p < 0.01, ^***^p < 0.001. AGA, appropriate for gestational age; ARI, Acute respiratory infections; BMI, Body mass index; SGA, Small for gestational age.

Children born SGA and preterm had significantly (*p* < 0.001) lower mean birth weight and length compared to the AGA group (Table 1). The mean gestational age was 34.9, 39.5, and 40.5 weeks for preterm, AGA and SGA infants, respectively. Among the preterm group, the majority were classified as late preterm infants (78% between 34- < 37 weeks). The rate of early initiation of breastfeeding was lowest in the preterm group (*p* < 0.05), but no differences were observed for exclusive breastfeeding or child dietary diversity among the three groups. Child morbidity also did not differ among the three groups at 1y and 2y, but it was highest in the SGA group at 6–7y.

### Postnatal growth in preterm, SGA, and AGA children

Table 2 displays average weight, height and growth velocity for the three groups of children across different time periods. Compared to the term AGA group, those born preterm were lighter and shorter at birth (3.2 kg vs. 2.9 kg and 49.3 cm vs. 48.7 cm, respectively) and at 3 months of age (5.3 kg vs. 5.1 kg and 57.5 cm vs.56.9 cm, respectively) but had similar anthropometric measurements thereafter. In contrast, the SGA group consistently demonstrated lower weight and length compared to the AGA group in all periods.

**Table 2:** Postnatal weight (kg), length/height (cm), and growth velocity in weight (kg/month) and height (cm/month) in preterm, SGA, and AGA children.

	AGA	Preterm	SGA
Weight, mean (SD)			
Birth	3.18 (0.35)^a^	2.91 (0.44)^b^	2.60 (0.30)^c^
3m	5.25 (0.68) ^a^	5.08 (0.75)^b^	4.79 (0.70)^c^
6m	7.75 (0.93) ^a^	7.79 (0.92) ^a^	7.11 (0.81)^c^
12m	8.88 (1.02)^a^	8.76 (0.98)^a^	8.16 (0.96)^c^
18m	9.73 (1.05)^a^	9.60 (0.98)^a^	8.99 (0.96)^c^
24m	10.90 (1.15)^a^	10.63 (1.08)^b^	10.09 (1.06)^c^
6–7y	18.96 (3.15)^a^	18.87 (2.87)^a^	17.79 (3.53)^c^
10–11y	30.55 (7.51)^a^	31.26 (7.09)^a^	28.32 (7.30)^c^
Weight increase (gr), mean (SD)			
Birth -3m	1.10 (0.26)^a^	1.11 (0.24)^a^	1.10 (0.27)^a^
3–6m	0.50 (0.14)^a^	0.54 (0.15)^b^	0.47 (0.13)^a^
6–12m	0.23 (0.10)^a^	0.21 (0.11)^b^	0.21 (0.11)^a,b^
12–18m	0.15 (0.10)^a^	0.16 (0.10)^a^	0.14 (0.08)^a^
18–-24m	0.17 (0.09)^a^	0.14 (0.08)^b^	0.16 (0.09)^a,b^
0–2y	0.32 (0.44)^a^	0.32 (0.43)^a,c^	0.31 (0.42)^c^
2–6/7y	0.15 (0.05)^a^	0.15 (0.03)^a^	0.15 (0.05)^a^
6/7–10/11y	0.26 (0.11)^a^	0.27 (0.11)^a,c^	0.24 (0.10)^c^
Length/Height, mean (SD)			
Birth	49.26 (2.83)^a^	48.67 (2.87)^a^	47.03 (3.12)^c^
3m	57.47 (2.52) ^a^	56.89 (2.76) ^b^	56.23 (2.60) ^b^
6m	67.29 (2.55)^a^	66.93 (2.63)^a^	65.68 (2.38)^c^
12m	72.88 (2.61)^a^	72.51 (2.75)^a^	71.12 (2.72)^c^
18m	77.66 (2.67)^a^	77.16 (2.37)^a^	75.82 (2.48)^c^
24m	83.16 (3.07)^a^	82.59 (2.99)^a^	81.25 (3.09)^c^
6–7y	113.82 (5.15) ^a^	113.46 (4.76) ^a,b^	112.20 (5.85) ^b^
10–11y	135.47 (6.81)^a^	135.88 (6.43)^a^	133.43 (6.61)^c^
Length/height increase (cm), mean (SD)			
Birth -3m	4.38 (1.68)^a^	4.33 (1.57)^a^	4.59 (1.76)^a^
3–6m	1.98 (0.46)^a^	2.02 (0.50)^a^	1.95 (0.49)^a^
6–12m	1.16 (0.40)^a^	1.16 (0.34)^a^	1.11 (0.37)^a^
12–18m	0.89 (0.34)^a^	0.92 (0.35)^a^	0.86 (0.29)^a^
18–24m	0.77 (0.26)^a^	0.76 (0.28)^a^	0.77 (0.29)^a^
0–2y	1.40 (0.15)^a^	1.40 (0.15)^a^	1.41(0.18)^a^
2–6/7y	0.59 (0.06)^a^	0.59 (0.06)^a^	0.59 (0.08)^a^
6/7y–10/11y	0.49 (0.07)^a^	0.49 (0.08)^a^	0.48 (0.08)^a^

AGA, appropriate for gestational age; SGA, small for gestational age; SD, Standard deviation. Labeled values in a row without a common superscript letter differ. *P* < 0.05, using ANOVA test followed by Bonferroni *post hoc* correction for pair-wise comparison of means.

Weight velocity was highest during the first 3 months of life (~1.1 kg/month), lowest between ages 2 and 6 years (~0.15 kg/month) and increased between age 6–11 years (0.24–0.27 kg/month). Compared to AGA infants, preterm infants gained more weight during the first 6 months of life (4.88 kg for preterm vs. 4.57 kg for AGA) but had had similar increases in weight and length after 6 months of age. In contrast, SGA children had slower weight gain in the first 2 years of life and between 6 and 10 y when compared to AGA children. We did not find any significant differences in length/height velocity among the three groups, but it was highest in the first 3 months of life (~4.4 cm/months) and reduced gradually thereafter (~0.5 cm between 6–10y).

**Figure 2:**
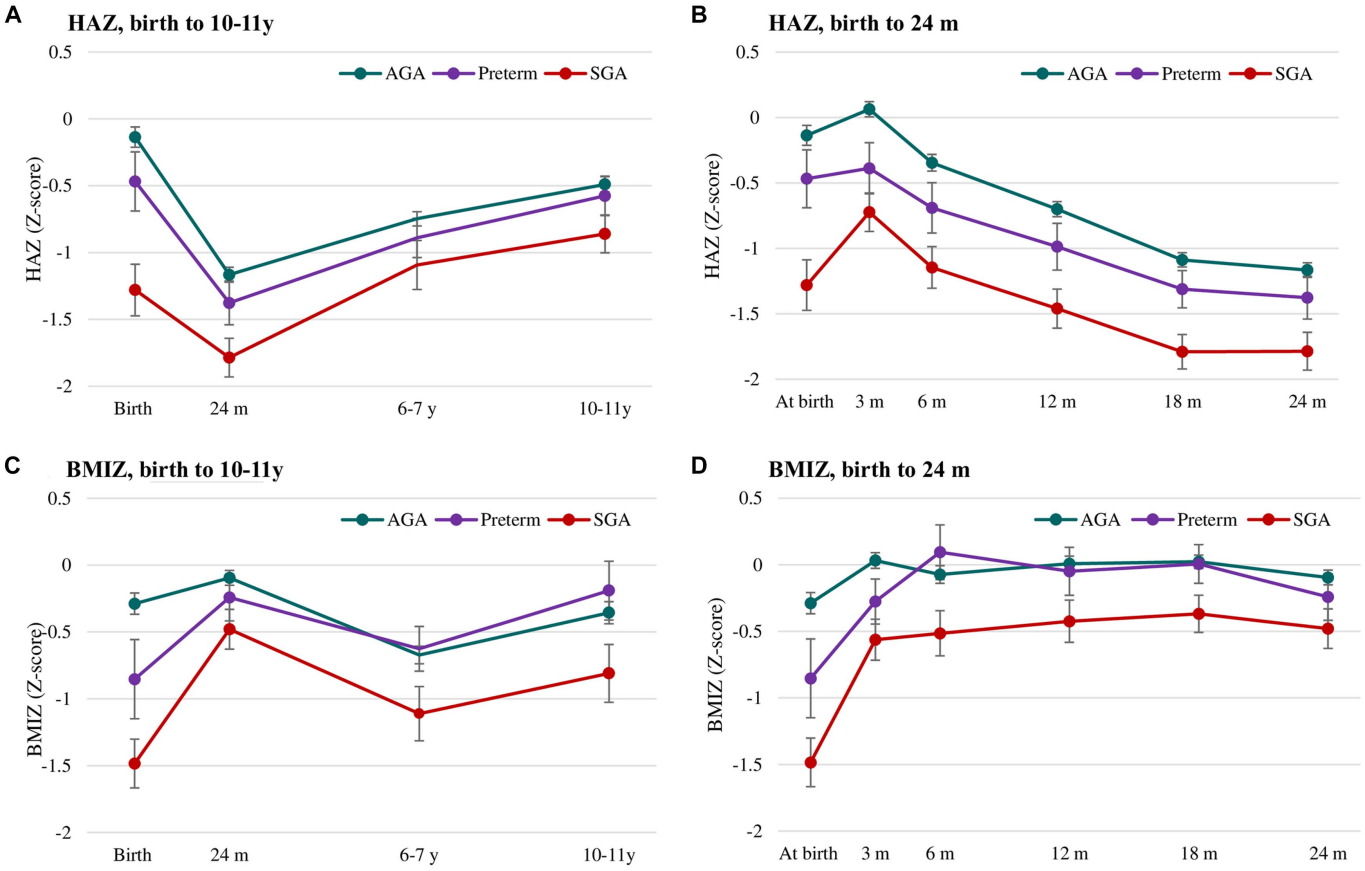
Postnatal growth of HAZ **(A,B)** and BMIZ **(C,D)** in preterm, SGA and AGA children. Values are mean (95% CIs). AGA, appropriate for gestational age; SGA, small for gestational age; HAZ, Height for age z-score; BMIZ, Body mass index z-score.

Comparisons of mean HAZ and BMIZ scores by birth status during the first 10 years of life are shown in Figure 2 and Supplementary Table S2. There were notable differences in HAZ among the three groups. Compared to the AGA group, the preterm group had lower HAZ until age 2 y while the SGA groups had lower HAZ in all periods (Figures 2A,B). HAZ exhibited increasing trends in all three groups in the first 3 months, then declining until 24 months and increasing trends thereafter. For BMIZ, the preterm group had lower BMIZ in the first 3 months, but similar BMIZ to that of the AGA group in later life (Figures 2C,D). In contrast, the BMIZ of SGA groups remained consistently lower in all periods compared to that of AGA group.

### Association of prematurity and SGA on the risk of childhood and adolescent under- and over-nutrition

**Figure 3:**
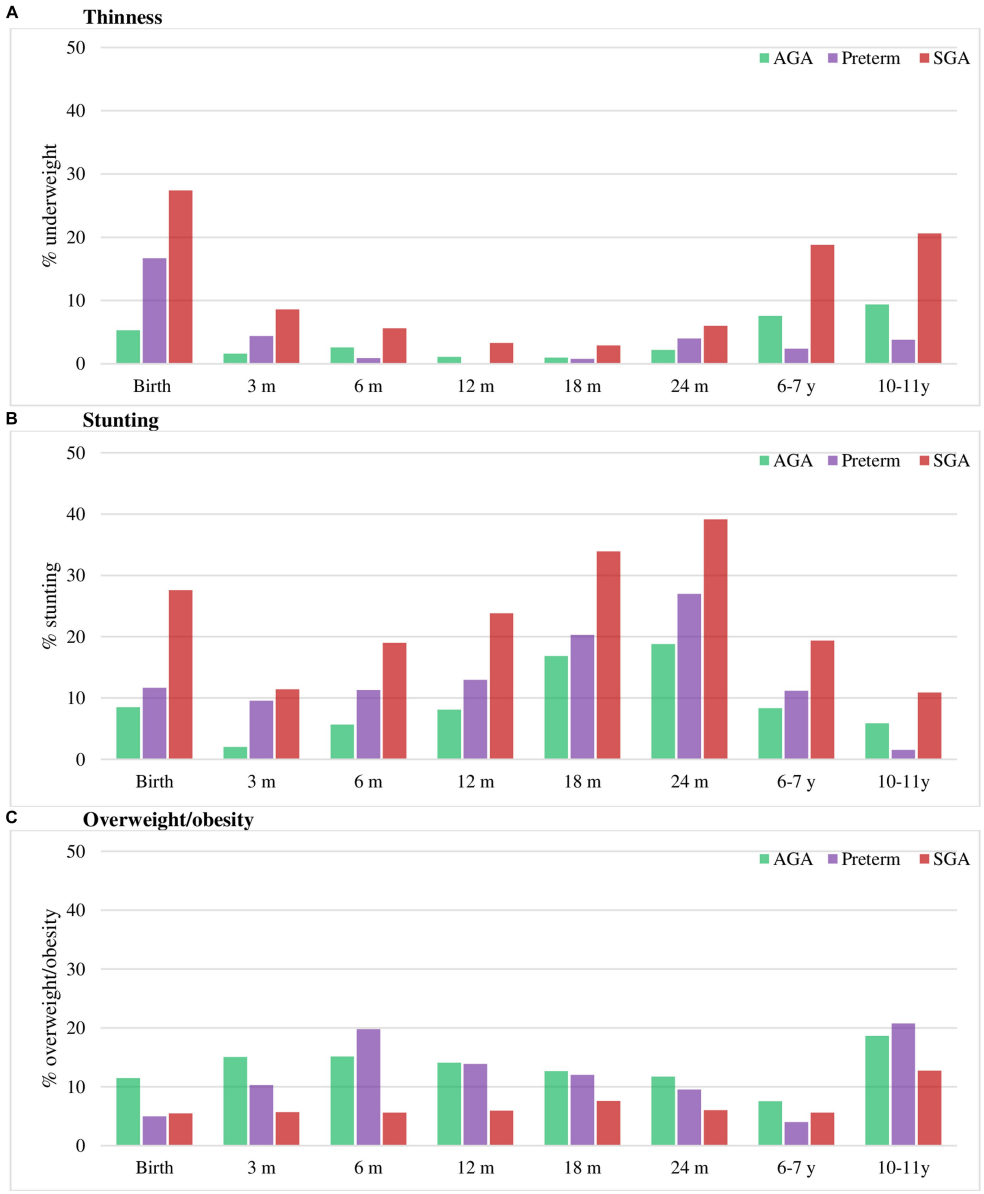
Thinness **(A)**, stunting **(B)** and overweight/obesity **(C)** in preterm, SGA and AGA children. Values are percentage. AGA, appropriate for gestational age; SGA, small for gestational age. Stunting was defined as HAZ below −2 Z-score. Thinness was defined as BMIZ below −2 Z-score and overweight/obese as BMIZ above 1 Z-score (38).

The nutritional status of three groups is shown in Figure 3 and Supplementary Table S3. At birth, the prevalence of underweight and stunting was highest in SGA (22 and 28%, respectively), followed by preterm (10 and 12%, respectively) and AGA group (0.3 and 9%, respectively). Compared to the AGA group, the rates of thinness and stunting was consistently higher in SGA group in all periods, but only significantly higher in preterm group in the first 3 months for thinness and first 6 months for stunting. The rate of overweight/obesity did not significantly differ between preterm and AGA group, but was lower in SGA compared to AGA group in the first year of life.

Multivariable regression analysis adjusted for potential confounding factors at child, maternal and household levels showed that compared to those born AGA, SGA infants were 2.5–2.6 times higher risk of thinness at 2y and 6y; and this risk reduced to 1.6 times at 10–11y (Table 3). The risk of stunting was also 2.4 and 2.3 times higher in SGA than AGA group at ages 2y and 6–7y, respectively, with no differences at 10y. Although preterm children had higher rates of thinness and stunting at 2y compared to AGA children, these differences were not statistically significant. We did not find any differences in overweight/obesity by birth phenotype (Table 4). Additionally, the risk of stunting was reduced among taller mothers, and children born to mothers with low BMI were about 2 times higher risk of thinness (Table 3), whereas those born to mothers with high BMI were 6.5 and 3.6 times higher risk of overweight/obesity at 6–7y and 10–11y, respectively (Table 4).

**Table 3:** Association between birth conditions (preterm, SGA, and AGA) and thinness and stunting in childhood and early adolescence.

	2 years		6–7 years		10–11 years	
	Thinness	Stunting	Thinness	Stunting	Thinness	Stunting
	AOR[95%CI]	AOR[95%CI]	AOR[95%CI]	AOR[95%CI]	AOR[95%CI]	AOR[95%CI]
Study group						
AGA (ref)						
Preterm	1.42[0.46, 4.37]	1.43[0.87, 2.34]	0.24^+^[0.06, 1.02]	1.38[0.68, 2.82]	0.27^*^[0.08, 0.87]	0.14^+^[0.02, 1.05]
SGA	2.45^+^[0.99, 6.06]	2.40^***^[1.54, 3.76]	2.57^**^[1.43, 4.61]	2.25^**^[1.24, 4.08]	1.56[0.87, 2.79]	1.49[0.72, 3.10]
Female	0.64[0.31, 1.30]	0.74^+^[0.55, 1.00]	0.95[0.61, 1.50]	1.20[0.78, 1.85]	1.06[0.69, 1.61]	1.74^*^[1.00, 3.01]
Early initiation of breast feeding	1.47[0.71, 3.03]	1.46^*^[1.08, 1.98]	0.84[0.53, 1.32]	0.92[0.60, 1.42]	1.02[0.67, 1.57]	1.21[0.70, 2.10]
Child morbidity						
ARI	1.77[0.85, 3.67]	0.74^*^[0.55, 1.00]	1.36[0.86, 2.15]	1.14[0.73, 1.76]	1.07[0.70, 1.64]	1.52[0.88, 2.65]
Diarrhea	1.17[0.34, 4.06]	0.97[0.53, 1.77]	1.53[0.68, 3.45]	0.72[0.29, 1.78]	1.71[0.78, 3.72]	0.89[0.29, 2.71]
Maternal factors						
Age	1.04[0.95, 1.13]	0.98[0.94, 1.02]	1.02[0.96, 1.08]	0.99[0.93, 1.04]	0.96[0.91, 1.02]	1.00[0.93, 1.07]
Mother height	0.98[0.91, 1.05]	0.88^***^[0.85, 0.91]	1.03[0.99, 1.08]	0.86^***^[0.81, 0.90]	1.03[0.99, 1.08]	0.87^***^[0.82, 0.92]
BMI						
Normal BMI (18.5–<23) (ref)						
Low BMI (<18.5)	1.38[0.66, 2.87]	1.10[0.79, 1.52]	1.92^**^[1.21, 3.05]	1.47[0.93, 2.32]	2.11^***^[1.38, 3.22]	1.55[0.89, 2.70]
High BMI (≥23)	0.58[0.08, 4.50]	0.84[0.42, 1.65]	0.42[0.10, 1.81]	0.54[0.16, 1.82]	0.19[0.03, 1.44]	1.00[1.00, 1.00]
Education						
Primary school (ref)						
Secondary school	0.34^*^[0.13, 0.87]	0.82[0.50, 1.35]	0.40^**^[0.20, 0.80]	1.27[0.58, 2.78]	0.58[0.29, 1.17]	0.96[0.39, 2.32]
High school	0.30^*^[0.10, 0.97]	0.61^+^[0.34, 1.08]	0.35^*^[0.15, 0.79]	0.60[0.24, 1.53]	0.59[0.27, 1.29]	0.60[0.20, 1.73]
College or higher	0.20^+^[0.04, 1.01]	0.38^*^[0.17, 0.85]	0.73[0.26, 2.10]	0.41[0.11, 1.50]	0.67[0.22, 2.01]	0.22^+^[0.04, 1.30]
Occupation (farmer)	1.24[0.91, 1.68]	1.04[0.88, 1.23]	0.93[0.70, 1.23]	0.97[0.75, 1.25]	0.98[0.77, 1.26]	1.12[0.85, 1.48]
≥ 2 child	0.93[0.19, 4.47]	1.05[0.54, 2.07]	0.78[0.30, 2.03]	1.21[0.43, 3.40]	0.71[0.29, 1.74]	1.48[0.33, 6.72]
Household factors						
Socio-economic status						
High (ref)						
Middle	1.06[0.43, 2.61]	1.35[0.91, 2.01]	1.28[0.70, 2.35]	0.67[0.38, 1.18]	0.97[0.54, 1.74]	0.79[0.38, 1.66]
Low	0.82[0.33, 2.07]	1.11[0.75, 1.66]	1.18[0.63, 2.18]	0.60^+^[0.34, 1.05]	1.44[0.82, 2.51]	1.04[0.52, 2.09]

Significant difference: ^+^*p* < 0.1, ^*^p < 0.05, ^**^ *p* < 0.01, ^***^p < 0.001. All estimates were from multiple logistic regression adjusted for potential confounding factors including at child (sex, age, early initiation of breast feeding, morbidity), maternal levels (age, height, BMI, education, occupation, number of children) and household socio-economic status. AGA, Appropriate for gestational age; AOR, Adjusted Odds Ratio; ARI, Acute respiratory infections; BMI, Body mass index; CI, Confidence interval; SGA, small for gestational age. Stunting was defined as HAZ below -2 Z-score. Thinness was defined as BMIZ below -2 Z-score (38).

**Table 4:** Association between birth conditions (preterm, SGA, and AGA) and overweight/obesity in childhood and early adolescence.

	Overweight/obesity at 2 years	Overweight/obesity at 6–7 years	Overweight/obesity at 10–11 years
	AOR[95%CI]	AOR[95%CI]	AOR[95%CI]
Study group			
AGA (ref)			
Preterm	0.82[0.40, 1.70]	0.31[0.07, 1.34]	1.52[0.88, 2.60]
SGA	0.59[0.26, 1.32]	0.98[0.37, 2.61]	0.81[0.43, 1.53]
Female	0.71^+^[0.48, 1.04]	0.84[0.49, 1.45]	0.55^***^[0.39, 0.78]
Early initiation of breast feeding	1.01[0.68, 1.49]	0.80[0.46, 1.38]	0.67^*^[0.48, 0.95]
Child morbidity			
ARI	0.89[0.61, 1.32]	0.93[0.54, 1.60]	0.89[0.64, 1.26]
Diarrhea	0.77[0.32, 1.85]	1.16[0.39, 3.42]	0.93[0.45, 1.95]
Maternal factors			
Age	1.00[0.95, 1.05]	0.93^*^[0.86, 1.00]	0.98[0.94, 1.02]
Mother height	0.97^+^[0.93, 1.01]	0.99[0.93, 1.05]	1.00[0.96, 1.03]
BMI			
Normal BMI (18.5–<23) (ref)			
Low BMI (<18.5)	0.59^*^[0.37, 0.96]	0.69[0.35, 1.37]	0.84[0.57, 1.25]
High BMI (≥23)	1.71[0.87, 3.37]	6.51^***^[3.17, 13.38]	3.59^***^[2.00, 6.42]
Education			
Primary school (ref)			
Secondary school	1.14[0.54, 2.39]	0.64[0.23, 1.81]	0.61[0.34, 1.11]
High school	1.43[0.64, 3.20]	1.25[0.42, 3.69]	0.74[0.38, 1.43]
College or higher	0.98 [0.36, 2.69]	1.35 [0.34, 5.34]	1.16 [0.50, 2.67]
Occupation (farmer)	1.05[0.87, 1.27]	1.12[0.88, 1.41]	1.27^**^[1.08, 1.49]
≥ 2 child	1.42[0.54, 3.77]	0.52[0.21, 1.29]	0.58[0.30, 1.15]
Household factors			
Socio-economic status			
High (ref)			
Middle	0.80[0.48, 1.31]	0.66[0.34, 1.28]	1.04[0.67, 1.60]
Low	0.85[0.51, 1.40]	0.62[0.31, 1.26]	0.80[0.51, 1.27]

Significant difference: ^+^*p* < 0.1, ^*^p < 0.05, ^**^p < 0.01, ^***^p < 0.001. All estimates were from multiple logistic regression adjusted for potential confounding factors including at child (sex, age, early initiation of breast feeding, morbidity), maternal levels (age, height, BMI, education, occupation, number of children) and household socio-economic status. AGA, Appropriate for gestational age; AOR, Adjusted Odds Ratio; ARI, Acute respiratory infections; BMI, Body mass index; CI, Confidence interval; SGA, small for gestational age. Overweight/obese was defined as BMIZ above 1 Z-score (38).

## Discussion

In this well-designed prospective study, we found that preterm children experienced rapid weight gain in the first 6 months of life when compared to AGA children, with no differences in linear growth rates. They continued to be shorter until age 2y but caught up to the AGA group at 6 years. SGA children also had lower HAZ and BMIZ, and although the differences decreased over time, they did not catch-up completely with AGA children by age 10–11 y. Children born SGA were also 2–3 times more likely to be undernourished in all periods compared to AGA infants, but the risk reduced when children get older. No associations were found between preterm or SGA with overweight /obesity.

Our findings on rapid postnatal growth in the first 6 months of life among preterm infants are aligned with previous studies, although the magnitude of catch-up growth is different. For example, one study in Italy showed 80% catch-up growth in weight and length at 3 and 6 months (17), while another study in China showed 26–28% catch-up growth between 0 and 6 months (14). Some possible reasons for rapid postnatal growth in the first few months of life include the high proportion (78%) of late preterm infants (34–37 weeks) in our sample who are less likely to develop serious diseases and able to grow better after birth, and the continuity of the velocity of intrauterine growth (which is greater than at any time in postnatal life). However, after the first 6 months, infants born preterm in our study still had lower HAZ at 1 and 2 years of age, which is in contrast to other studies that observed significant level of catch-up growth in the first 2 years of life (8, 11, 16, 43). Findings on long-term growth patterns are limited and mixed, with some studies showing catch-up growth at 8y (21) or 10y (22), while other research reporting that preterm children remained shorter and lighter than AGA children throughout childhood (9). It is important to note that those studies are from high income countries. As one of very few studies on long-term growth patterns in low-and middle-income countries, our findings indicated that the catch-up growth among preterm children occurred at 6y and maintained at 10y. Potential mechanisms that explained for difference in growth of preterm children include differences in infant and young child feeding practices (20), neonatal morbidity (21) or endocrine regulation (plasma IGF-I and IGFBP-3 concentrations) of postnatal growth (9).

In our study, children born SGA had lower HAZ and BMIZ, as well as higher risk of undernutrition compared to those born AGA, although the differences decreased with age. These findings are in contrast with several studies that suggest full catch-up growth among SGA children may occur within 2 years of life (11, 12, 16), but consistent with a few other studies showing that SGA group had smaller height at age 12 years compared to AGA group in USA (21), or no catch up growth at 10 years among children born SGA in the Dutch nationwide prospective study (22). SGA children often exposed to early environmental stress involving variable nutritional restriction *in utero*, thus it has been hypothesized that these children may suffer from relative resistance to a number of hormones (such as GH, IGF-I, and insulin) or the defect of receptors (IGF-I receptor defect or a post–receptor-mediated defect) which may be the basis for an alteration of endocrine programming resulting (44). Therefore, being born with SGA is not only associated with postnatal growth failure but also possibly an increased risk of metabolic syndrome and cardiovascular diseases in later life.

Being born preterm or SGA has been identified as risk factors for overweight/obesity in later life due to the *in utero* imprinting which result in resistance to multiple hormones (45). Additionally, rapid catch-up growth in these children placed them at higher risks of childhood obesity (16, 23). In our study, however, we did not observe any association between preterm or SGA and overweight /obesity at 6–7y or 10 y. Whether these associations may happen in later life will require longer follow-up because preterm birth has been found associated with higher fat mass in males at age 30 years (46), and faster weight gain in the first 3 months was positively associated with body fat percentage and waist circumference at 21 years (24). Potential negative long-term effects of catch-up growth and programming of body composition remains critical area of research to inform strategies on optimal growth for small and vulnerable newborns (47).

Key strengths of our study include well-designed longitudinal study that span from preconception, during pregnancy, birth, childhood and early adolescent in low-resource settings with a high follow-up rate of 87% at the age of 10 y. The rich data on multiple assessments of growth at different ages with a standardized methodology allowed us to examine the growth pattern of preterm and SGA children during and beyond the first 1,000 days. Finally, the availability of data at age 6–7 y and 10–11 years is very useful for planning and designing intervention strategies; the 6–7 years represents the beginning of the school age years when children are exposed to new and challenging environments while the 10–11 years represents early adolescents which is a sensitive transition time with higher demands for nutrition for faster growth. This study, however, has some limitations. First, gestational age was estimated using the last menstrual period (LMP) which may not be as accurate as those estimated from ultrasound measure during the first trimester. However, LMP in our study was assessed prospectively by village health workers during biweekly home visits which can reduce recall errors. We also had ultrasound measurements in a subsample and have previously published that that the median gestational age estimated by ultrasound during second trimester was close to that of LMP (274 days vs. 276 days) (31). Another limitation is the small number of preterm SGA infants (*n* = 9) and early preterm births (*n* = 33) who may be at higher risk compared to preterm AGA infants. Finally, although we had data on maternal characteristics such as age, height, weight, and BMI, we lacked paternal heights and weights, and it would have been useful to also have biomarker data such as lipids and glucose to evaluate future metabolic risk (24, 25). Future studies that include biomarker data with more follow-up through the adolescent years will be valuable to advance current understanding of the mechanism and growth pattern by birth condition later in life.

In conclusion, despite global commitments in the last 30 years, limited progress has been made to reduce the burden of adverse birth outcomes such as preterm delivery and fetal growth restriction (3), Recent recommendations propose a new definition for the “small vulnerable newborn,” bringing preterm birth, SGA and low birth weight together, for a better focus on the health of mothers and fetuses (1). Efforts to reduce the burden of these conditions is needed with studies that continue to document the adverse consequences that are preventable through investments to improve access to timely and high-quality care for women of reproductive age and their offspring from birth through adulthood.

## Data availability statement

The raw data supporting the conclusions of this article will be made available by the authors, without undue reservation.

## Ethics statement

The studies involving humans were approved by the Ethical Committee of Institute of Social and Medicine Studies in Vietnam and Emory University’s Institutional Review Board, Atlanta, Georgia, United States. The studies were conducted in accordance with the local legislation and institutional requirements. Written informed consent for participation in this study was provided by the participants’ legal guardians/next of kin.

## Author contributions

PTN: Conceptualization, Formal analysis, Investigation, Methodology, Writing – original draft. PHN: Conceptualization, Funding acquisition, Investigation, Methodology, Supervision, Writing – original draft, Writing – review & editing. LT: Data curation, Methodology, Visualization, Writing – review & editing. LK: Data curation, Formal analysis, Methodology, Visualization, Writing – review & editing. SN: Resources, Supervision, Writing – review & editing. MY: Methodology, Writing – review & editing. UR: Funding acquisition, Investigation, Methodology, Writing – review & editing.
