# Supplementary material for: Growth patterns of preterm and small for gestational age children during the first 10 years of life
Source: Front Nutr. 2024 Feb 26;11:1348225. doi: 10.3389/fnut.2024.1348225 (PMC10925699; doi:10.3389/fnut.2024.1348225)
Supplement: Supplementary file 1 [file Data_Sheet_1.docx]

**Supplemental Table 1. Characteristics of analytic sample and missing sample at 10y**

|  | **Analytic sample**  **(n=1367)** | **Missing sample**  **(n=203)** |
| --- | --- | --- |
| **Mother characteristics** |  |  |
| Mother’s age at birth, y | 26.0 (4.3) | 25.1 (4.2)* |
| Mother education, % |  |  |
| Completed primary school | 8.0 | 7.9*** |
| Completed secondary school | 56.4 | 38.4 |
| Completed high school | 25.2 | 28.1 |
| College or higher | 10.4 | 25.6 |
| Occupation, % |  |  |
| Work as farmers | 18.3 | 34.5*** |
| Other jobs | 81.7 | 65.5 |
| Parity, % |  |  |
| 1 child | 4.9 | 9.3* |
| ≥2 children | 95.1 | 90.7 |
| Mother’s height, cm | 152.7 (5.1) | 153.0 (4.9) |
| BMI |  |  |
| Mother underweight (BMI<18.5), % | 69.3 | 67.2 |
| Mother overweight (BMI ≥23), % | 30.7 | 32.8 |
| **Household characteristics** |  |  |
| Socio-economic status, % |  |  |
| High | 35.2 | 25.2** |
| Middle | 33.2 | 31.7 |
| Low | 31.6 | 43.1 |
| **Child characteristics** |  |  |
| Birthweight, g | 3085.7 (437.3) | 3071.4 (415.8) |
| Birth length, cm | 48.9 (3.0) | 49.1 (2.7) |
| Gestational age, weeks | 39.2 (2.0) | 39.2 (2.0) |
| Sex (female), % | 50.4 | 41.9* |
| Child feeding, % |  |  |
| Early initiation of breastfeeding | 52.7 | 44.2* |
| Exclusive breastfeeding | 59.6 | 55.5 |
| Dietary diversity at 1y | 69.3 | 72.8 |
| Child morbidity at 1y, % |  |  |
| ARI | 59.1 | 59.6 |
| Diarrhea | 13.6 | 14.0 |
| Child morbidity at 2y, % |  |  |
| ARI | 49.5 | 48.3 |
| Diarrhea | 5.8 | 6.9 |
| Child morbidity at 6-7y, % |  |  |
| ARI | 30.5 | 29.1 |
| Diarrhea | 1.3 | 0.1 |

Values are percentage or mean (SD). Significant difference: ^*^ *p* < 0.05, ^**^ *p* < 0.01, ^***^ *p* < 0.001. ARI: Acute respiratory infections, BMI: Body mass index HAZ: Height for age zscore, WAZ: weight for age zscore

**Supplemental Table 2: Postnatal growth of HAZ and BMIZ in preterm, SGA and AGA children**

|  | **AGA**  **(1)** | **Preterm**  **(2)** | **SGA**  **(3)** | **Difference**  **(2-1)** | **Difference**  **(3-1)** | **Difference**  **(3-2)** |
| --- | --- | --- | --- | --- | --- | --- |
| **HAZ, mean (SD)** |  |  |  |  |  |  |
| Birth | -0.14 (1.28) | -0.47 (1.24) | -1.30 (1.27) | -0.33^*^ [-0.57,-0.09] | -1.16^***^ [-1.37,-0.95] | -0.83^***^ [-1.13,-0.53] |
| 3 m | 0.06 (1.02) | -0.39 (1.16) | -0.72 (0.99) | -0.45^***^ [-0.63,-0.27] | -0.79^***^ [-0.95,-0.62] | -0.34^**^ [-0.57,-0.11] |
| 6 m | -0.35 (1.04) | -0.69 (1.01) | -1.15 (0.97) | -0.35^**^ [-0.55,-0.14] | -0.80^***^ [-0.98,-0.62] | -0.45^***^ [-0.71,-0.20] |
| 12 m | -0.70 (0.95) | -0.99 (0.95) | -1.46 (0.94) | -0.29^**^ [-0.48,-0.10] | -0.76^***^ [-0.92,-0.60] | -0.47^***^ [-0.71,-0.24] |
| 18 m | -1.09 (0.94) | -1.31 (0.84) | -1.79 (0.88) | -0.22^*^ [-0.39,-0.06] | -0.70^***^ [-0.85,-0.55] | -0.48^***^ [-0.69,-0.27] |
| 24 m | -1.17 (0.95) | -1.38 (0.94) | -1.79 (0.95) | -0.21^+^ [-0.39,-0.04] | -0.62^***^ [-0.77,-0.47] | -0.41^***^ [-0.63,-0.19] |
| 6-7 y | -0.75 (0.91) | -0.89 (0.84) | -1.09 (1.18) | -0.14 [-0.32,0.03] | -0.35^***^ [-0.50,-0.19] | -0.20^+^ [-0.42,0.02] |
| 10-11y | -0.49 (0.96) | -0.58 (0.86) | -0.86 (0.92) | -0.09 [-0.26,0.09] | -0.37^***^ [-0.53,-0.22] | -0.28^*^ [-0.50,-0.07] |
| **BMIZ, mean (SD)** |  |  |  |  |  |  |
| Birth | -0.29 (1.35) | -0.85 (1.65) | -1.48 (1.19) | -0.57***[-0.82,-0.31] | -1.20*** [-1.42,-0.97] | -0.63***[-0.95,-0.31] |
| 3 m | 0.03 (1.03) | -0.28 (1.01) | -0.56 (1.04) | 0.31^**^ [-0.49,-0.12] | -0.59^***^ [-0.76,-0.43] | -0.29^*^ [-0.52,-0.06] |
| 6 m | -0.07 (1.08) | 0.09 (1.08) | -0.51 (1.03) | 0.17 [-0.05,0.38] | -0.44^***^ [-0.63,-0.25] | -0.61^***^ [-0.88,-0.34] |
| 12 m | 0.01 (0.93) | -0.05 (0.96) | -0.42 (0.99) | -0.06 [-0.24,0.13] | -0.43^***^ [-0.59,-0.27] | -0.38^**^ [-0.61,-0.14] |
| 18 m | 0.02 (0.87) | 0.01 (0.86) | -0.37 (0.93) | -0.02 [-0.17,0.14] | -0.39^***^ [-0.53,-0.25] | -0.37^***^ [-0.57,-0.18] |
| 24 m | -0.10 (0.95) | -0.24 (1.00) | -0.48 (0.98) | -0.15 [-0.32,0.03] | -0.38^***^ [-0.54,-0.23] | -0.24^*^ [-0.46,-0.02] |
| 6-7 y | -0.67 (1.12) | -0.63 (0.95) | -1.11 (1.31) | 0.05 [-0.16,0.25] | -0.44^***^ [-0.63,-0.25] | -0.49^***^ [-0.75,-0.22] |
| 10-11y | -0.36 (1.38) | -0.19 (1.28) | -0.81 (1.42) | 0.17 [-0.09,0.42] | -0.45^***^ [-0.68,-0.23] | -0.62^***^ [-0.94,-0.30] |

Significant difference: ^+^ *p* < 0.1, ^*^ *p* < 0.05, ^**^ *p* < 0.01, ^***^ *p* < 0.001. AGA: Appropriate for gestational age, SGA: Small for gestational age, HAZ: Height for age zscore, BMIZ: Body mass index zscore, SD: Standard deviation **Supplemental Table 3: Child under- and over-nutrition in preterm, SGA and AGA children**

|  | **AGA**  **(1)** | **Preterm**  **(2)** | **SGA**  **(3)** | **Difference**  **(2-1)** | **Difference**  **(3-1)** | **Difference**  **(3-2)** |
| --- | --- | --- | --- | --- | --- | --- |
| **Thinness, (%)** |  |  |  |  |  |  |
| Birth | 5.3% | 16.7% | 27.4% | 11.38[6.20,16.57] | 22.2 [17.64,26.67] | 10.77 [4.29,17.25] |
| 3 m | 1.6% | 4.4% | 8.6% | 2.81 [0.02,5,65] | 6.97[4.43,9.51] | 4.16 [0.57,7.74] |
| 6 m | 2.6% | 0.9% | 5.6% | -1.69 [- 5.01,1.62] | 2.99 [0.08,5.91] | 4.69 [0.52,8.86] |
| 12 m | 1.1% | 0.0% | 3.3% | -1.14 [- 3.39,1.09] | 2.17[0.24,4.09] | 3.31 [0.51 – 6.11] |
| 18 m | 1.0% | 0.8% | 2.9% | -0.29[- 2.27,1.69] | 1.88 [0.11,3.66] | 2.17 [-0.33 – 4.67] |
| 24 m | 2.2% | 4.0% | 6.0% | 1.73[- 1.32,4.78] | 3.78 [1.08,6.49] | 2.05 [-1.78 – 5.89] |
| 6-7 y | 7.6% | 2.4% | 18.8% | -5.15 [- 10.23,-0.00] | 11.19 [6.63,15.74] | 16.35 [9.92 – 22.77] |
| 10-11y | 9.4% | 3.8% | 20.6% | -5.57 [-11.05,-0.00] | 11.18 [6.25,16.11] | 16.75[9.84 -23.67] |
| **Stunting, (%)** |  |  |  |  |  |  |
| Birth | 8.52 | 11.67 | 27.61 | 3.14 [-2.68,8.95] | 19.08 [14.01,24.16] | 15.94 [8.67,23.21] |
| 3 m | 2.02 | 9.56 | 11.43 | 7.53 [4.19,10.88] | 9.41 [6.41, 12.40] | 1.87 [-2.35,6.09] |
| 6 m | 5.67 | 11.32 | 19.01 | 5.65 [0.40,10.90] | 13.34 [8.74,17.95] | 7.69 [1.09,14.30] |
| 12 m | 8.10 | 12.96 | 23.84 | 4.86 [-1.09,10.81] | 15.74 [10.61, 20.87] | 10.88 [3.45,18.30] |
| 18 m | 16.84 | 20.30 | 33.92 | 3.46 [-3.55,10.47] | 17.08 [10.80,23.35] | 13.61 [4.77,22.47] |
| 24 m | 18.80 | 26.98 | 39.16 | 8.18 [0.65,15.72] | 20.36 [13.69,27.03] | 12.17 [2.70,21.64] |
| 6-7 y | 8.36 | 11.20 | 19.37 | 2.84 [-2.65, 0.83] | 11.01 [6.09,15.93] | 8.18 [1.23,15.12] |
| 10-11y | 5.88 | 1.54 | 10.91 | -4.34 [-8.68,-0.00] | 5.03 [1.13,8.94] | 9.37 [3.89,14.85] |
| **Overweight/obesity, (%)** |  |  |  |  |  |  |
| Birth | 11.48 | 5.00 | 5.49 | -6.48 [-12.17,-0.78] | -5.99 [-10.95,-1.03] | 0.48 [-6.63,7.60] |
| 3 m | 15.08 | 10.29 | 5.71 | -4.79 [-10.84,1.27] | -9.37 [-14.78,-3.95] | -4.58 [-12.23,3.07] |
| 6 m | 15.15 | 19.81 | 5.63 | 4.66 [-2.34,11.68] | -9.52 [-15.68,-3.36] | -14.18 [-23.01,-5.35] |
| 12 m | 14.11 | 13.89 | 5.96 | -0.22 [-6.91,6.47] | -8.15 [-13.91,-2.39] | -7.93 [-16.27,0.41] |
| 18 m | 12.67 | 12.03 | 7.60 | -0.64 [-6.48,5.20] | -5.07 [-10.30,0.16] | -4.43 [-11.80,2.95] |
| 24 m | 11.73 | 9.52 | 6.02 | -2.20 [-7.94,3.53] | -5.70 [-10.78,-0.63] | -3.50 [-10.71,3.71] |
| 6-7 y | 7.55 | 4.00 | 5.62 | -3.55 [-8.28,1.17] | -1.93 [-6.17,2.31] | 1.63 [-4.36,7.61] |
| 10-11y | 18.66 | 20.77 | 12.73 | 2.11 [-4.91,9.13] | -5.93 [-12.49,0.39] | -8.04 [-16.90,0.83] |

Significant difference: ^+^ *p* < 0.1, ^*^ *p* < 0.05, ^**^ *p* < 0.01, ^***^ *p* < 0.001. AGA: Appropriate for gestational age, SGA: Small for gestational age
